# Dr. Soumya Swaminathan: A Pioneer in Global Health and Equity

**DOI:** 10.7759/cureus.74225

**Published:** 2024-11-22

**Authors:** Aishwarya Kalluri, Kristal De La Cruz Quezada, Carolina Rodriguez, Donna Musegue, Kenneth A Quezada, Krishn B Patel, Asha Plamoottil, Nadiya A Persaud, Latha Ganti

**Affiliations:** 1 Research, Orlando College of Osteopathic Medicine, Winter Garden, USA; 2 Research, Orlando College of Osteopathic Medicine, Orlando, USA; 3 Primary Care, Orlando College of Osteopathic Medicine, Winter Garden, USA; 4 Emergency Medicine & Neurology, University of Central Florida, Orlando, USA; 5 Medical Science, The Warren Alpert Medical School of Brown University, Providence, USA

**Keywords:** covid, historical vignette, hiv, innovator, soumya swaminathan, tuberculosis

## Abstract

Dr. Soumya Swaminathan, a renowned Indian physician and scientist, revolutionized medical practices through her significant contributions to tuberculosis and human immunodeficiency virus research, yielding a lasting impact on global health. As the former Chief Scientist of the World Health Organization, her committed approach to healthcare has been instrumental in linking scientific research and evidence to policy development. Swaminathan’s work has saved countless lives and shaped global tuberculosis control strategies, particularly in resource-limited areas. As a dedicated physician, researcher, and international health leader, her unwavering commitment to science has left a lasting impact on modern medicine. This historical vignette explores her early life, her journey into the medical field, and her transformative contributions to medicine and public health.

## Introduction and background

Dr. Soumya Swaminathan was raised in India in a family rooted in science and community service [1]. Inspired by her upbringing, she developed a passion for medicine and public health from a young age. Driven by her dedication to providing equitable access to healthcare from the earliest and most vulnerable stages of life, she chose to pursue a career in pediatrics [2]. Swaminathan’s lifelong work reflects her commitment to bridging the gap between science and healthcare on both a local and global scale.

Early life

Born on May 2, 1959, in Chennai, India, Dr. Soumya Swaminathan was raised in a family that valued science, education, and social responsibility. Her mother, Mina Swaminathan, was an influential social worker who specialized in preschool education, specifically for underprivileged children. Her father, Dr. M.S. Swaminathan, was an Indian geneticist globally recognized for his leading role in India’s “Green Revolution” [1]. Widely regarded as the “Father of the Green Revolution,” Dr. M.S. Swaminathan’s pioneering contributions to sustainable agriculture played a pivotal role in crop development and harvesting high-yielding varieties of wheat and rice during India's post-independence period of food insecurity [3,4]. He planted seedlings in the fields of poor farmers using sustainable farming practices, efficiently improving agricultural productivity and boosting food security [3]. He is credited with saving millions from starvation through his dedicated efforts to increase agricultural productivity and production in the late 20th century [3]. A lifelong believer and advocate for the power of science, he applied a holistic approach to agriculture that preserved biodiversity while increasing employment opportunities by empowering female farmers in rural areas [3,4]. Dr. Soumya Swaminathan spent much of her childhood accompanying her father to work, recalling vivid memories from her trips to the “gamma garden.” There, gamma rays were used to alter the genetic composition of plants, promoting desirable traits that improved growth [4]. Raised in a nurturing and supportive environment, she was exposed to early conversations about science, agriculture, and social justice. These greatly influenced her career trajectory. 

Medical education and training

The seed of Dr. Soumya Swaminathan's passion for medicine was sown during an influential project she completed as a part of her scholarship awarded at the All-India Science Talent Competition [4]. This experience solidified her interest and laid the foundation for her future career in medicine. Under the mentorship of her professor, Archana Sharma, in Kolkata, India, she studied chromosomal defects with specific focus on a case involving a young girl with Turner Syndrome [2,4]. Dr. Swaminathan's fascination grew, inspiring her to study genetics at Hindu College and explore the realm of research [4]. However, her path to medical school was far from straightforward. At first, Dr. Swaminathan set out to study Zoology, aspiring to become a veterinarian. However after conversations with third- and fourth-year medical students, she decided to take the entrance exam for the Armed Forces Medical College, shifting her focus to medicine [2,4]. Although initially waitlisted, Dr. Swaminathan was later invited for an interview and ultimately accepted to this prestigious institution. 

Upon completing her medical education at the Armed Forces Medical College in 1980, Dr. Soumya Swaminathan specialized in pediatrics at the All India Institute for Medical Sciences (AIIMS) in New Delhi, one of India’s most esteemed medical schools. Here, she not only trained in the technical aspects of medicine, but was also exposed to the challenges of providing healthcare in resource-limited settings [4]. Before the academic year at AIIMS commenced, Dr. Swaminathan completed an internship at the military hospital located in Safdarjung, New Delhi [4]. This internship required home visits in the undeveloped Palam village, where Dr. Swaminathan was confronted by the high prevalence of widespread unscientific biases and superstitions related to childcare. She recalled witnessing parents feeding tea and honey to their newborns, unaware of the health implications, and rejecting colostrum due to misconceptions about its impurity and potential harm [4]. These eye-opening observations reinforced the importance of education and public health for Dr. Swaminathan, a commitment that would later resonate throughout her future global health initiatives. 

Following the completion of her specialization at AIIMS in 1982, Dr. Swaminathan still longed to conduct research. This motivated her to explore opportunities outside India. She embarked on a medical fellowship in neonatology and pediatric pulmonology at the Children’s Hospital Los Angeles, where she refined her research skills and expertise under the guidance of her mentor Professor Thomas G Keens [4]. During this time, Dr. Swaminathan published six influential academic papers focused on pediatric pulmonology, and later continued her research experience as a fellow at the University of Leicester in the United Kingdom [2,4]. 

Dr. Swaminathan’s return to India in 1992 marked a turning point in her career. Energized with motivation and determination, she assumed the role of research fellow at the National Institute for Research in Tuberculosis (NIRT) in Chennai, India [4]. During that time, India was battling the rising prevalence of human immunodeficiency virus (HIV) further complicated by its co-infection tuberculosis (TB), an epidemic sweeping both Asia and Africa [2,4]. Dr. Swaminathan was entrusted with the significant responsibility of leading NIRT’s HIV research and response efforts, a role which she approached with poise and effectiveness [4].

## Review

TB-HIV research

Dr. Swaminathan’s contributions to medicine and public health are both extensive and impactful. While at NIRT, Dr. Swaminathan led numerous research projects that significantly advanced the global understanding of TB and HIV, specifically in children. Her meticulous research highlighted the challenges associated with the early diagnosis of HIV infections in infants [2].

Dr. Swaminathan established India’s impactful TB-HIV research program from the ground up. As HIV became more widespread at the beginning of the 21st century, confusion and hysteria surrounding the disease intensified. The stigma associated with TB and HIV led to reluctance among healthcare workers to treat affected patients, exacerbating the epidemic amidst India’s limited resource bank [2]. Dr. Swaminathan tirelessly researched the medical and socioeconomic determinants related to this epidemic, spending extensive time working with marginalized communities and raising awareness about the inequities they faced [2,4]. Her influential publication, *Pediatric Tuberculosis: Global Overview and Challenges*, highlighted the urgent need for tailored diagnostic criteria and treatment methods for children infected with both TB and HIV. She shed light on the rapid progression from infection to disease in children and described the challenges in diagnosis caused by non-specific symptoms and limited bacteriological confirmation [5]. Furthermore, her work that identified drug-resistant forms of TB emphasized the increased vulnerability of children, raising awareness about preventive methods and the alarming rates of pediatric infections [6]. Dr. Swaminathan’s effective leadership at the NIRT played an influential role in shaping the global epidemiological response and strengthened the strategies for the prevention, diagnosis, and treatment of HIV and TB infections, specifically amongst the pediatric population.

Leadership

From 2009 to 2011, Dr. Swaminathan’s legacy and leadership continued as she served as the Coordinator of the United Nations International Children's Emergency Fund (UNICEF)/United Nations Development Programme (UNDP)/World Bank/World Health Organization (WHO)'s Special Program for Research and Training in Tropical Diseases (TDR) in Geneva [7]. In this role, she led the research and training initiatives aimed at addressing tropical diseases, employing a targeted public health approach that prioritized diseases disproportionately affecting low- and middle-income countries [7].

From 2009 to 2017, Dr. Swaminathan expanded her leadership, serving as the Director General of the Indian Council of Medical Research (ICMR), and from 2015 to 2017, as the Secretary of the Department of Health Research [8]. In these roles, she sought to integrate scientific principles and evidence into policy [8]. She worked to implement key initiatives such as the *National Ethical Guidelines for Biomedical Research Involving Children*, which provided protection to pediatric participants in biomedical studies, and the *Treatment Guidelines for Antimicrobial Use in Common Syndromes*, which provided evidence-based guidelines aimed at reducing antimicrobial resistance [9,10].

WHO

In 2017, Dr. Swaminathan joined the WHO as the Deputy Director-General and was subsequently appointed as the organization’s first Chief Scientist in 2019. While much of her tenure involved combating the effects of the COVID-19 pandemic, she also played a significant role in setting the standards for vaccines and drugs, developing treatment guidelines, and advocating for equitable access to the vaccine among low- and middle-income countries [2]. During the pandemic, Dr. Swaminathan led a comprehensive daily analysis of global COVID-19 research [11,12]. She incorporated political, economic, social, and cultural factors into the development of effective, evidence-based policies and guidelines to guide healthcare professionals globally [11], while simultaneously accelerating vaccine development [12]. These efforts included developing treatment guidelines through extensive collaboration and continuous, rigorous research while allowing treatments to remain flexible and updated as new research findings emerged [13].

Health education and the present day

**Figure 1:**
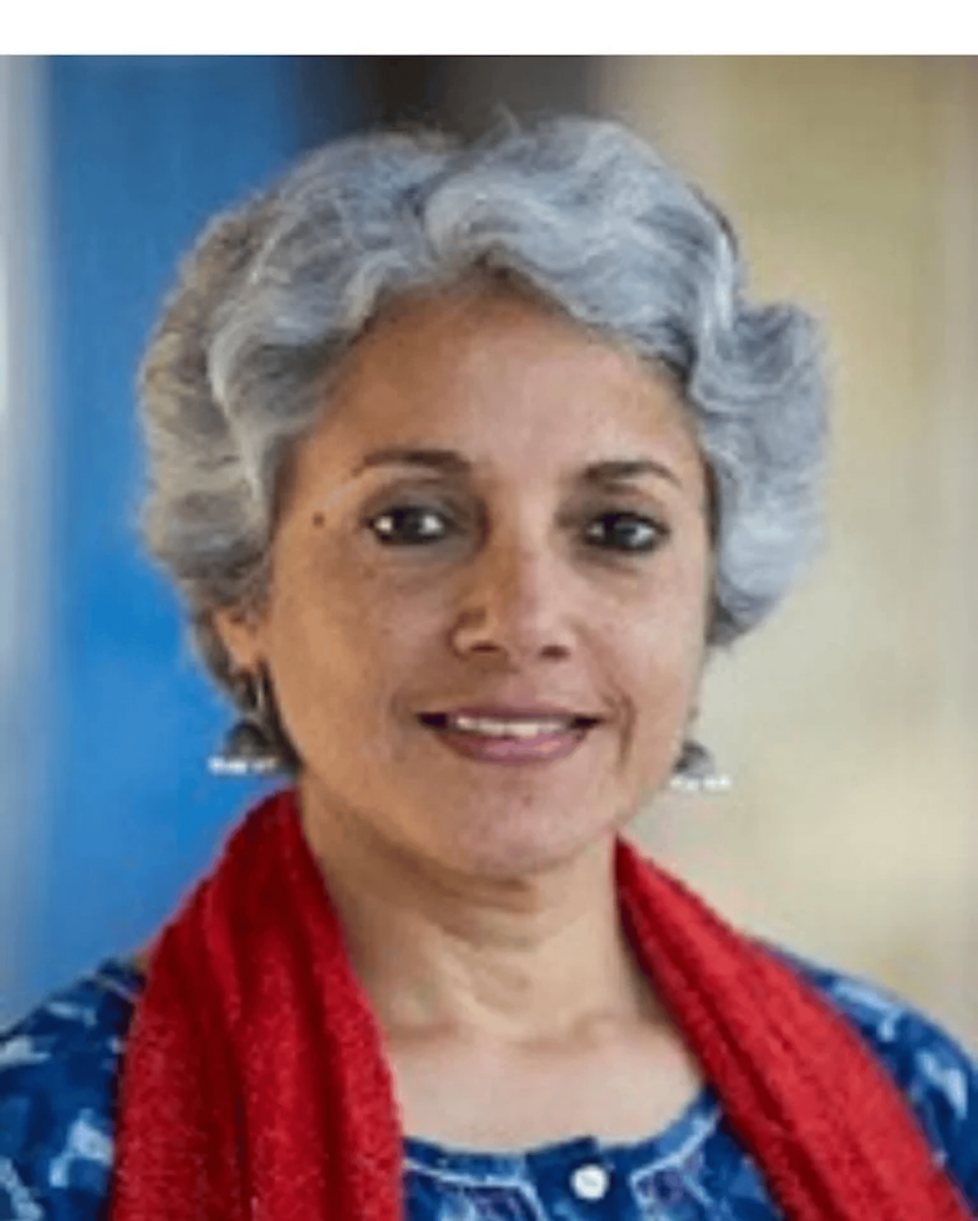
Dr. Soumya Swaminathan Image from Tufts University Office of the Vice-Provost for Research.

In 2009, Dr. Swaminathan became an Adjunct Clinical Associate Professor in the Department of Public Health and Community Medicine at Tufts University (Figure1), where she brought her extensive expertise in medicine, public health, and clinical research [14]. She educated and inspired many aspiring healthcare professionals to pursue meaningful careers in these essential fields, fueling innovation groundbreaking research. 

Building upon her impactful work at the WHO where she played a key role in policy-making, Dr. Swaminathan now works on public health guideline development with a primary focus on research. She promotes the use of research and the incorporation of digital technologies to ensure all departments are up-to-date with the advancements in medicine [14]. She educates and helps bridge the health literacy gaps that are apparent in today’s population, specifically in the marginalized communities [14]. Dr. Swaminathan aims to educate others on mental health, as the prevalence of mental health conditions has increased dramatically after the pandemic, mostly among young people [14]. She also influences the community by bringing awareness to the topic of climate crisis and educating individuals on the ways to battle these problems in today’s generation [14].

In 2022, Dr. Soumya Swaminathan stepped down from her position at the WHO to return to her homeland, India, to work further on public health initiatives. She assumed the position of Chairperson at the M.S. Swaminathan Research Foundation, an initiative founded by her father to advance clinical research and sustainability [8]. 

On May 28, 2024, she was honored by McGill University with an honorary Doctor of Science degree in health sciences for her lifetime contribution to medicine and global health. Dr. Swaminathan was among the 10 exceptional individuals to receive this honorary degree, McGill’s highest honor [15,16].

**Figure 2:**
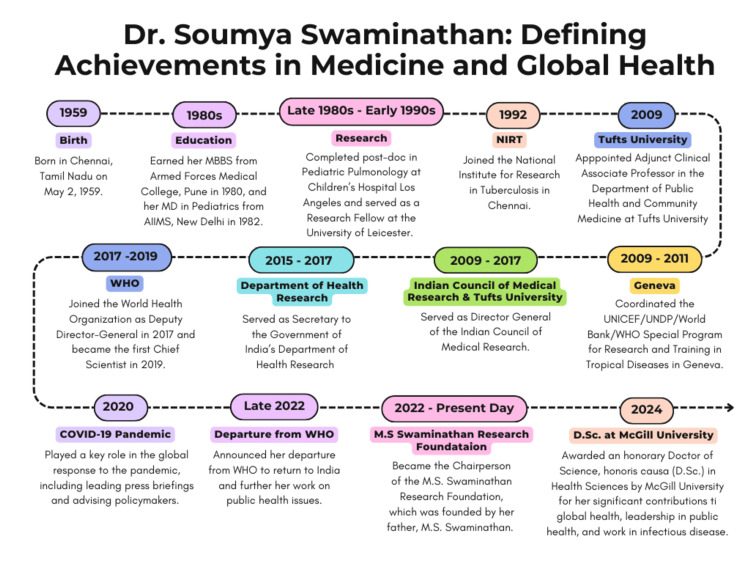
Dr. Soumya Swaminathan: Defining achievements in medicine and global health Infographic designed by Nadiya A Persaud on Canva. Software: Canva Pro (Canva Inc., Perth, Australia) MBBS: Bachelor of Medicine and Bachelor of Surgery; MD: Doctor of Medicine; AIIMS: All India Institute of Medical Sciences Delhi; WHO: World Health Organization; UNICEF: United Nations International Children's Emergency Fund; UNDP: United Nations Development Programme

Dr. Soumya Swaminathan's unwavering, lifelong commitment to advancing public health through compassionate clinical practice, rigorous clinical research, evidence-based policy, and effective leadership has profoundly improved global health outcomes, leaving a lasting legacy that continues to influence healthcare today (Figure 2).

## Conclusions

Dr. Swaminathan’s analytical approach to TB, HIV, and COVID-19 laid the foundation for global health standards, remarkably reducing the spread of infectious diseases. Her efforts to decrease the stigma amongst marginalized communities have fostered greater access to healthcare, encouraging these populations to seek treatment without fear of discrimination and significantly improving public health outcomes. With nearly 400 publications indexed in PubMed, many featured in prestigious journals such as The Lancet and The New England Journal of Medicine, Dr. Swaminathan's extensive research continues to advance medical knowledge, fueling progress in global healthcare. Dr. Soumya Swaminathan's legacy continues to shape more inclusive, evidence-based, and effective healthcare systems, resonating through present-day advancements and paving the way for future generations.
